# Sport and exercise medicine consultants are reliable in assessing tendon neovascularity using ultrasound Doppler

**DOI:** 10.1136/bmjsem-2017-000298

**Published:** 2018-02-07

**Authors:** James Watson, Robert M Barker-Davies, Alexander N Bennett, Daniel T P Fong, Patrick C Wheeler, Mark Lewis, Craig Ranson

**Affiliations:** 1 Academic Department of Military Rehabilitation, Defence Medical Rehabilitation Centre, London, UK; 2 Cardiff School of Sport, Cardiff Metropolitan University, Cardiff, UK; 3 National Centre for Sport and Exercise Medicine, School of Sport Exercise and Health Sciences, Loughborough University, London, UK; 4 Faculty of Medicine, National Heart and Lung Institute, Imperial College, London, UK; 5 Athlete Health Department, English Institute of Sport, Manchester, UK

**Keywords:** tendinopathy, achilles, ultrasound

## Abstract

**Objective:**

Several lower limb tendinopathy treatment modalities involve identification of pathological paratendinous or intratendinous neovascularisation to target proposed co-location of painful neoneuralisation. The ability to reliably locate and assess the degree of neovascularity is therefore clinically important. The Modified Ohberg Score (MOS) is frequently used to determine degree of neovascularity, but reliability has yet to be established among Sport and Exercise Medicine (SEM) consultants. This study aims to determine inter-rater and intra-rater reliability of an SEM consultant cohort when assessing neovascularity using the 5-point MOS.

**Method:**

Eleven participants (7 male and 4 female) provided 16 symptomatic Achilles and patella tendons. These were sequentially examined using power Doppler (PD) enabled ultrasound (US) imaging by 6 SEM consultants who rated neovascular changes seen using the MOS. Representative digital scan images were saved for rescoring 3 weeks later. Inter-rater and intra-rater reliability of the MOS was examined using intraclass correlation coefficient (ICC) and Kappa Agreement scores.

**Results:**

Neovascular changes were reported in 65.6% of 96 scans undertaken. ICC for inter-rater reliability was 0.86 and Fleiss Kappa 0.52. ICC for intra-rater reliability was 0.95 and Weighted Kappa 0.91.

**Conclusions:**

Neovascular changes were present in two-thirds of symptomatic tendons. Excellent SEM consultant inter-rater and intra-rater reliability was demonstrated. These findings support the use of PD-enabled US to assess neovascularity by appropriately experienced SEM consultants. Furthermore, future interventional research using a similarly experienced SEM consultant cohort can be undertaken with assurance that assessment of neovascularity will be reliable.

**What are the new findings?**

- Appropriately trained Sport and Exercise Medicine consultants have excellent inter-rater and intra-rater reliability using power Doppler enabled ultrasound to assess neovascularity in chronic Achilles and patellar tendinopathy.
- Previously, such results have only been demonstrated among sonographers and radiologists.
- Neovascularity is present in two-thirds of chronic Achilles and patellar tendinopathies.

## Introduction

Incidence and prevalence of Achilles and patellar tendinopathies are increased in physically active populations.^1–3^ During the progression of tendinopathic disease, the formation and ingrowth of neovascular structures into the tendon is a widely recognised pathological hallmark.^4–7^ The role of neovascularisation within tendinopathy is the source of significant interest regarding its contribution to the pathophysiology of the injured and healing tendon.^6 8^ It may also be associated with neoneuralisation^9^ exacerbated by nociceptive stimuli such as glutamate^10^ and substance P.^11^ This provides an explanation as to why the point of ingress of vessels into the tendon is often associated with the site of maximal pain on palpation.^12^ Neovascularisation is a normal physiological response in early repair, and its continued presence is likely to be a result of prolonged overload that delays the healing timeline.^13^ Association between neovascularity and pain and function has been shown in small interventional case and longitudinal studies^14–17^ yet refuted in Randomised Controlled Trial (RCT)^18^ and only weakly correlated in a larger prospective cohort.^7^ This supports the theory that neovascularity may be more a sign of chronicity than severity. Polidocanol^14^ and high volume image-guided injections (HVIGI)^19^ target the presence of vessels in order to address this proposed pain stimulus.^14 19^ To that end, the ability to reliably locate and quantify vascularity is clinically relevant.

The hypoechoic areas seen in tendinopathy on gray-scale ultrasound (US) offer limited information which can be enhanced by the use of power Doppler (PD) to identify hyperaemic neovasculature.^7 10 14 20 21^ In clinical practice, there have been 3 methods of assessing the degree of neovascularisation. The first is a simple statement of whether vessels are either absent or present in the tendon structure.^8^ Depending on the significance of degree of vascularity as discussed above, the utility of such binary grading is likely to be limited. A second system involves determining the PD signal surface area by pixel count. Although a seemingly robust method of capturing a quantifiable measure of neovascularity, these authors recognise that interpretation of findings in clinical practice will be both machine and operator setting dependent.^22 23^ A third method was pioneered by Ohberg and Alfredson^14^ who undertook PD US examination of symptomatic Achilles tendons to understand the extent of neovascularity and locate target areas for sclerosing polidocanol injections. In this study, an experienced sonographer assessed neovascularisation as 0, 1+, 2+, 3+ or 4+. In the absence of vessels, the tendon scored a 0. A score of 1+ was ascribed if the tendon showed 1–2 small vessels primarily around the anterior surface of the tendon. Scores of 2+, 3+ or 4+ were given if the vessel contained 2, 3 or more than 4 vessels respectively throughout its structure. They later adapted this score for use in patellar tendinopathy with the score of 1+ referring to 1–2 vessels mostly in the posterior portion of that tendon.^16^

Sengkerij *et al*^21^ undertook an inter-rater reliability study in which a cohort of radiologists undertook PD US examinations of symptomatic and asymptomatic Achilles tendons and rated the neovascularisation referring to the system described above as the Modified Ohberg Score (MOS). Of the 50 tendons examined in the study, each underwent scans by 2 of 8 radiologists. Excellent reliability was found with absolute agreement of 62% and an intraclass correlation coefficient (ICC) of 0.85. Sunding *et al*^24^ used a more qualitative 4-point MOS for neovascularity: 0, None; 1, Mild; 2, Moderate; 3, Severe. They therefore used Kappa in their analysis of 27 Achilles and 26 patella tendons finding good inter-rater agreements of 0.63 and 0.70 respectively using 2 sonographers.

US scanning is a common skill used frequently in clinical practice, yet there are no data to support its reliability among Sport and Exercise Medicine (SEM) consultants. This study aims to contribute to future research by establishing reliability among SEM consultants when assessing tendon neovascularity using the 5-point MOS.

## Method

Using a power of 80% (β-level=0.8) and an α-level of 0.05 anticipating good to excellent reliability (P_0_>0.7 to P_1_=0.9), the calculations of Walter *et al*^25^ were used to estimate the requirement for at least 5 raters (n) of 10 participants (k).

## Participants

All participants gave written informed consent. Participants self-identified following advertisement for volunteers with Achilles or patella tendon pain. A subjective and objective assessment from an experienced physiotherapist (JW) against inclusion/exclusion criteria (table 1) was conducted consecutively. Participants were requested to avoid any heavy sporting or loading activity 24 hours prior to US examination.

**Table 1:** Inclusion and exclusion criteria

Inclusion criteria	Exclusion criteria
18–60 years of age Clinical findings of Achilles or patellar tendinopathy: Localised pain to tendon Pain/stiffness on waking Pain on commencing loaded activity Pain after activity Pain on mobilising after rest Symptoms within last 12 months lasting for period in excess of 2 months	Systemic condition causing inflammatory tendinopathy

Six SEM consultant raters were recruited to the study having completed professional training for a mean of 8 years (range 1–23 years). Four had undergone additional postgraduate training in musculoskeletal US, while the remainder had attained significant clinical experience in scanning both Achilles and patella tendons.

### Data collection

Prior to testing, the consultant raters received an instructional brief on the examination protocol which included standardised positioning of participants, surface pressure produced through the probe at the contact point, labelling of recorded scans and clarity as to the descriptions of the MOS (table 2). Participants were subjected to repeat PD US examination by all 6 raters.

**Table 2:** Description of the 5-point Modified Ohberg Score

Modified Ohberg Score	Description
0	No vessels
1+	One vessel anterior to the Achilles One vessel posterior to the patella tendon
2+	One to two vessels throughout the tendon
3+	Three vessels throughout the tendon
4+	Four or more vessels throughout the tendon

Scans were performed using a Logic 9 US Scanner (GE Medical Systems, Chalfont St Giles, UK) with a high frequency linear ML 6-15 MHz transducer (GE Medical Systems). Consultants oriented themselves to the tendon using standard gray-scale US to identify areas of the tendon to focus subsequent PD scanning. Once PD mode was engaged, the first consultant  when scanning a participant’s tendon set and standardised the PD gain used for the subsequent consultants. The first consultant to scan was varied for each patient to control any bias in setting of PD gain. Where possible, participants were scanned in series by all 6 consultants within the same session. To control for changes in tendon loading that may affect vessel signal, participants were instructed to avoid heavy sporting or loading activity 24 hours prior to repeat examinations.

**Figure 1:**
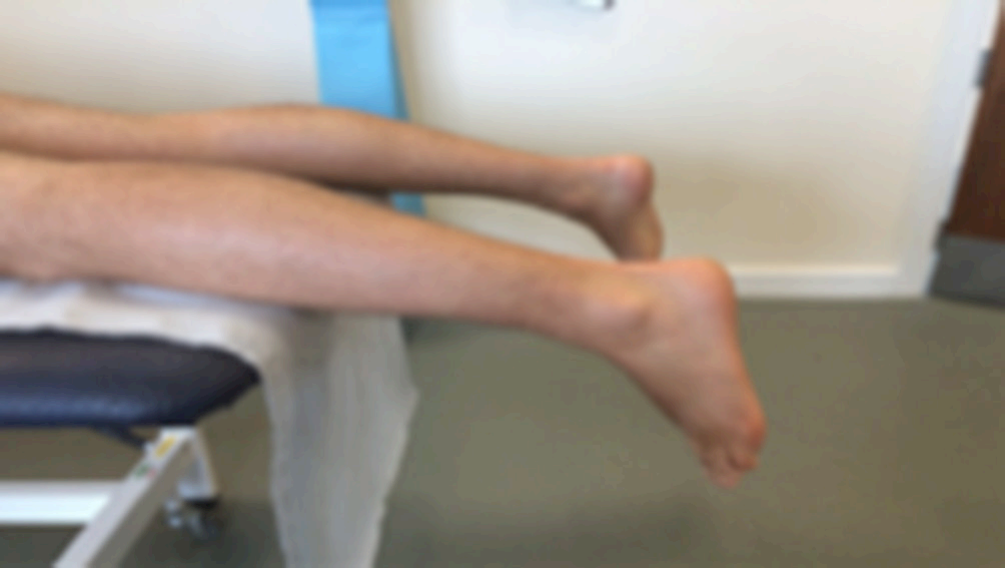
Standardised position for ultrasound imaging of the Achilles tendon.

**Figure 2:**
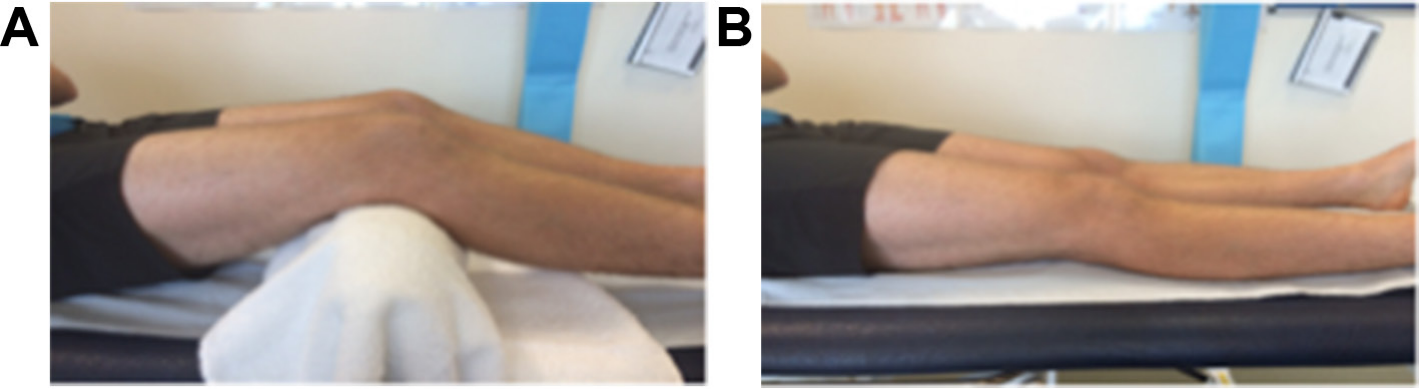
(A)Standardised position for US imaging of the patella tendon (position 1). (B) Standardised position for US imaging of the patella tendon (position 2). US, ultrasound.

To prevent vessel compression, positioning of the tendon was strictly controlled. Achilles tendons were scanned in the prone position with the foot in a relaxed non-dorsiflexed position over the end of the treatment couch (figure 1). Patella tendons were scanned in a standardised position of approximately 30° of knee flexion (figure 2A). Consultants were also permitted to scan in full knee extension (figure 2B) to remove any tension from the tendon.

### Data storage

Participants were assigned an anonymised unique identifier used to save images on the Logic 9 Scanner. Scans were saved once each consultant determined the MOS. Consultants were requested to save any scans that contributed to them formulating an overall MOS for the tendon. All MOS scores were recorded on paper retained out of sight of other raters. Three weeks after the initial examination, raters rescored the saved scans. Clinical information including previous MOS ratings was not available to consultant raters at any stage.

### Statistical analysis

Inter-rater and intra-rater reliability across the cohort of consultants was calculated using ICC and Kappa in IBM SPSS Statistics V.23 (IBM, Amonk, New York, USA) with a one-way random, single measures model. Fleiss defines an excellent ICC as higher than 0.75,^26^ a good ICC as 0.4–0.75 and poor if less than 0.4. For clinical investigations, an ICC of 0.60 has been described as the minimally acceptable level.^27^ Fleiss Kappa statistic was used to measure the level of agreement between raters, while a weighted Kappa was used to allow marginal agreement between individual raters’ baseline and follow-up scores.^28^ These were calculated using SPSS extension bundles. Both ICC and Kappa were used to provide comparability across the literature.

## Results

Eleven participants (7 males and 4 females with a mean age of 36.2 years (SD 6.25)) were recruited to the study and provided 16 symptomatic tendons. The mean duration of symptoms was 20.1 months (range 6–96 months).

### Presence of neovascularisation

Eight symptomatic Achilles and 8 patella tendons (n=16) were assessed by 6 consultant raters (K=6) resulting in 96 examinations. Neovascularisation was reported in 65.6% of scans and the MOS distribution is shown in table 3.

**Table 3:** Distribution of the Modified Ohberg Scores across all 96 examinations

Modified Ohberg Score	Number of scores recorded	Percentage of scores recorded
0	33	34.4
1+	23	23.9
2+	16	16.7
3+	5	5.2
4+	19	19.8

### Inter-rater reliability

In 6 tendons (37.5%), there was absolute scoring agreement across all raters, and a further 6 tendons had agreement from at least 4 of the 6 raters. This represents an agreement of 4 or more consultant raters in 75% of the participants. The ICC for inter-rater reliability of the MOS was 0.86 (95% CI, 0.76 to 0.94), illustrating excellent inter-rater reliability. The Fleiss Kappa agreement for inter-rater reliability was 0.52 (95% CI, 0.45 to 0.59). It should be noted the more modest result of Fleiss Kappa is not weighted and based on absolute agreement.

### Intra-rater reliability

Comparison of the agreement between baseline and follow-up scores can be found in table 4. Across raters absolute agreement between initial and follow-up scans occurred in 84 of 95 scans (one scan did not save), representing 88% of scans undertaken.

**Table 4:** Agreement between baseline and follow-up scoring

	Follow-up scan					
Baseline scan		0	1+	2+	3+	4+
	0	29	3	0	0	0
	1+	1	19	2	0	1
	2+	0	2	14	0	1
	3+	0	0	0	4	1
	4+	0	0	0	0	18

The ICC for intra-rater reliability within the cohort was 0.95 (95% CI, 0.92 to 0.97) illustrating excellent reliability of the MOS when reviewing scans. The weighted Kappa agreement score was 0.91 (95% CI 0.85 to 0.97) demonstrating excellent reliability.

## Discussion

This study has demonstrated excellent inter-rater reliability (ICC 0.86) of a cohort of SEM consultants when using the MOS to rate neovascularisation in Achilles and patella tendons. Furthermore, excellent intra-rater reliability (ICC 0.95) was demonstrated via review of saved scans. Neovascularisation was reported in 65.6% of the original scans in keeping with previous studies of Achilles and patellar tendinopathy.^21 29–31^ These results suggest that diagnostic US scoring of tendinopathic neovascularisation can be reliably employed in the SEM setting. The most important variable that will allow results to be extrapolated to a wider audience is the degree of clinical exposure to tendon assessment and ultrasonography clinicians have undertaken. All clinicians involved in this study described themselves as highly competent in the ultrasonography of Achilles and patella tendons, although this was not corroborated independently the results are comparable with those reported using radiologist or sonographer raters.^21 24^

In terms of distribution of MOS, it can be noted (table 3) that there was a lower number of Grade 3+ scores attributed to any of the primary scans undertaken. This is in keeping with the findings of Sengkerij and colleagues^21^ who found that only 9% of their scans received a 3+ score. An explanation for this is possibly that the raters find it easy to identify 1–2 vessels within the tendon structure, but once vascularity is more prevalent, it may be difficult to differentiate the existence of additional single vessels, the result being a lean towards a higher score of 4+. As the results of this study agree with those of Sengkerij *et al*,^21^ it may provide evidence to suggest that the 5-point scale is reduced to a 4-point scale, with the final score (grade 3) representing 3 or more vessels.

This study builds on the work of Sengkerij *et al*^21^ who did not attempt to control any of the operator variables once the PD setting was enabled. Of particular importance in this respect is the gain setting, which regulates the sensitivity of the system to flow.^32^ Essentially, too high a gain and the amount of random noise artefact in the form of colour foci in the image will increase, potentially affecting the clarity of colour that indicates vascular flow. In this study, the first consultant to scan a given participant was asked to select and set what they considered to be an appropriate gain, which other consultants would then use and this stayed consistent for that participant. As a result, the sensitivity of the system was standardised for each participant. Sengkerij *et al*^21^ used 2 radiologists each scanning session of a larger cohort (n=8). In such an uncrossed design, ICC does not readily deal with missing data. Using default SPSS settings with a one-way random ICC model cases that are not scanned by all raters would be excluded from the analysis. One solution to this would be to arrange data in 2 columns giving the appearance of a full data set from 2 raters exclusively. Collapsing data across empty columns and unequal proportions of scans undertaken by raters could overestimate the inter-rater ICC they reported of 0.85, though such limitations can only be assumed.

Arguably, the MOS is only a semiquantitative scale; therefore, this study has also used Fleiss and weighted Kappa. These favourable results compare well with Sunding *et al* who reported Kappas of 0.63 and 0.70 in Achilles and patellar tendinopathy, respectively using 2 independent sonographers.^24^ Weighted Kappa, which, like ICC, recognises marginal agreement and does not extend to comparison of more than 2 data sets. This presents a problem for Kappa analysis of more than 2 raters. The commonly used Fleiss Kappa result in this study should be interpreted differently from the other results because it can only reward absolute agreement. Light’s Kappa, which is the mean of all rating pairs,^33^ is a different solution yet not often reported. Therefore, the Fleiss Kappa has been presented in this study alongside an excellent inter-rater ICC, which has been able to provide context.

A strength of this study was the ability to recruit an appropriately powered number of consultant raters and where possible have them available for sequential scanning of all participants. Shoukri *et al*^27^ noted the difficulty in recruiting a number of suitably qualified specialists within the Hospital setting. While a large cohort of SEM consultants undertook ratings, short notice changes in availability were a limitation. The initial design aim for each participant to be scanned by 6 consultants sequentially in one session was achieved for 9 of 16 tendons. The remainder had to undertake subsequent scanning sessions (within a mean of 7.8 days). Boesen *et al*^22^ reported an increase in vessel appearance as an acute response to exercise, although postulate that this may be short-lived. As a result, those participants that were required to return for subsequent scans were requested to refrain from any activity that would either aggravate their symptoms or potentially make an acute change to the vessel appearance. This variable would have a potential to reduce consultant agreement on their primary scans of each patient, but despite this, excellent reliability was still demonstrated.

## Further research

The findings of this study have direct implications for future research and clinical practice. Primarily, a subcohort of the consultants in this study are also involved in a large RCT^34^ to investigate the effects of HVIGI (with and without steroid) on chronic Achilles and patella tendon pain. The neovessels found on scanning of the participants will be targeted by the injections delivered, and therefore this reliability study serves to provide assurance that the consultants are reliable when scoring neovasculature presence and extent. Furthermore, it can be extrapolated that consultants of similar experience to those in this study will provide reliable PD US assessments for future studies using the MOS, as either an outcome measure or target for intervention. In the clinical setting, this study provides evidence to support the use of diagnostic US in SEM clinical practice by proving reliability both in initial scanning and in the test-retest situation, such as follow-up scanning postintervention.

## Conclusion

This study demonstrated excellent inter-rater and intra-rater reliability of the scoring of neovasculature within tendon structure among SEM consultants. While this contributes to the clinical assessment of patients with tendon pain, it does not explain the role of neovasculature in the pathological process or as a source of pain in chronic tendinopathy. Well-controlled research is required to establish whether procedures targeting neovascularisation can effect improvements in validated pain and functional outcome measures.
